# Risk assessment and spatial-temporal distribution characteristics of nitrogen and phosphorus emissions from agricultural sources in the Yangtze River Economic Belt

**DOI:** 10.7717/peerj.20687

**Published:** 2026-02-05

**Authors:** Guang Wei Hu, Ying Xu, Yi Lan Ma

**Affiliations:** 1 School of Economics and Management, Dali University, Dali, China; 2 College of Urban and Environmental Science, Hunan University of Technology, Zhuzhou, China

**Keywords:** Yangtze River Economic Belt, Nitrogen and phosphorus pollution from agricultural sources, Spatiotemporal distribution, Geographic Information System (GIS)

## Abstract

Rapid socioeconomic development has intensified nitrogen and phosphorus pollution in the Yangtze River Economic Belt (YREB), posing severe threats to the water environment. Notably, agricultural sources have surpassed industrial sources to become the primary contributor to this pollution. To explore the spatiotemporal characteristics and impacts of agricultural nitrogen and phosphorus pollution in this region, this study constructed an improved output coefficient model. Using panel data from 11 provinces and municipalities in the YREB spanning 2005–2020, combined with Geographic Information System (GIS) spatial analysis tools, we calculated the pollution loads of agricultural nitrogen and phosphorus from three key sources: land use, rural domestic activities, and livestock and poultry breeding (LPB). The results show a consistent downward trend in total nitrogen (TN) and total phosphorus (TP) pollution loads from the three sources. Specifically, TN load decreased from 2,686.68 × 10^3^ tons/yr in 2005 to 2,035.67 × 10^3^ tons/yr in 2020, while TP load declined from 139.42 × 10^3^ tons/yr to 102.52 × 10^3^ tons/yr, with an average TN-to-TP pollution load ratio of approximately 19.40. GIS-generated spatial distribution maps confirm a widespread reduction in agricultural TN and TP pollution across the region. To effectively mitigate agricultural nitrogen and phosphorus pollution risks, we recommend strengthening research on fertilization techniques for major crops and exploring the establishment of centralized treatment stations for agricultural solid waste in regions within the YREB.

## Introduction

Amid accelerated industrialisation, the Yangtze River Economic Belt (YREB) has emerged as China’s most populous and economically dynamic inland economic zone. Agricultural nitrogen and phosphorus pollution has become the leading cause of land nitrogen and phosphorus enrichment and water eutrophication in the region. This issue is intertwined with ecological challenges such as land nutrient depletion and degradation of the natural environment supporting agricultural activities. Simultaneously, it triggers social problems including water eutrophication, environmental deterioration, and habitat destruction, which not only disrupt human activities but also threaten the sustainable development of the global economy and society.

In recent years, agricultural nitrogen and phosphorus pollution has attracted substantial research attention. Due to the universality, randomness, and detectability challenges of non-point source pollution, common estimation methods include the output coefficient method (Ma & Wang, 2021; Li et al., 2016), discharge coefficient method (Xie et al., 2019), and hydrological division method (Tu et al., 2012). Building on existing research highlighting the roles of policy and land use in agricultural pollution control (Liu, 2012), this study quantifies two key aspects: the spatial dominance of cropland changes in explaining pollution distribution variability across the YREB, and the relative contributions of three pollution sources (land use, livestock and poultry breeding (LPB), and rural domestic activities) to total pollution loads.

Among these methods, the output coefficient method is favored for its low parameter requirements, strong operability, and proven applicability and accuracy in estimating pollution loads in large watersheds, making it widely adopted in related studies. Research on pollution sources indicates that chemical fertilizers have the most significant impact on total nitrogen (TN) and total phosphorus (TP) pollution loads in the YREB (Zhu & Huang, 2022). In the Xinjiang River Basin, rural domestic activities and cropland are the main sources of TN, while rural domestic activities and LPB dominate TP pollution (Li et al., 2021). In the Huaihe River Eco-Economic Belt, chemical fertilizers, LPB, and aquaculture are the primary contributors to agricultural non-point source TN and TP pollution (Guo & Huang, 2021).

Regarding the spatiotemporal distribution of agricultural pollution, significant heterogeneity exists across different regions and river basins, influenced by factors such as local economic development levels, topography, rural domestic activities density, and livestock and aquaculture intensity (Guo & Huang, 2021; Zhu & Huang, 2022). Temporal variation studies show that non-point source pollution have shown that the pollution loads evolve over time (Ge et al., 2022), driven by economic development, urbanization, and agricultural policies, typically exhibiting an initial increase followed by a decrease. For example, research on non-point source pollution in Shandong Province (Duan, 2020) revealed distinct seasonal patterns in TN and TP pollution loads throughout the year.

In terms of spatial differentiation, non-point source pollution loads are closely associated with provincial administrative boundaries (Zhu & Huang, 2022), topographic features (Guo & Huang, 2021), watershed distributions (Hu et al., 2023; Ma et al., 2015), and reservoir systems (Chang, 2015), reflecting underlying natural conditions and socioeconomic environments. Importantly, the key driving factors of agricultural non-point source pollution vary significantly across these spatial units.

Taking land use, LPB, and rural domestic activities as evaluation units, this study establishes a risk assessment model to capture the overall impact of these three agricultural sources on the water environment. By analyzing the spatiotemporal distribution of TN and TP loads in different regions of the YREB using Geographic Information System (GIS), we aim to accurately identify pollution sources and formulate targeted policies—critical steps for mitigating agricultural nitrogen and phosphorus pollution risks in the region.

## Materials and Methods

### Study area

Anchored by the Yangtze River’s golden waterway, the YREB spans China’s eastern, central, and western regions, encompassing 11 provinces and municipalities: Shanghai, Jiangsu, Zhejiang, Anhui, Jiangxi, Hubei, Hunan, Chongqing, Sichuan, Guizhou, and Yunnan. It covers an area of approximately 2.05 million square kilometers, accounting for 21.35% of China’s total land area. In 2020, the total agricultural economic output value of the YREB reached 2.89 trillion yuan, representing 40.26% of the national total, with an urbanization rate of 63.22%.

In recent years, the water quality of the 11 provinces and municipalities has improved significantly: the proportion of sections with poor water quality decreased by 3.30 percentage points, while the proportion of excellent water quality sections increased by 13.80 percentage points. The operating cost of wastewater treatment facilities in the YREB has also risen annually, with a 30.20% increase in 2020 (Chen et al., 2022).

### Data

#### Data source

Economic, land use, livestock production, and rural domestic activities data were sourced from the “China Statistical Yearbook” (2006–2021) and the statistical yearbooks of the 11 provinces and municipalities in the YREB (Note: 2005 Data are from the 2006 yearbook, and 2020 data are from the 2021 yearbook). All data are publicly accessible on the National Data Website (https://data.stats.gov.cn/publish.htm?sort=1).

For LPB data, the number of livestock was determined based on their growth cycles: year-end cattle stocks represent annual breeding quantities, while year-end pig and sheep stocks indicate breeding quantities (Luo et al., 2015).

Annual statistics on year-end stocks (cattle, pigs, sheep) were obtained from provincial yearbooks, with adjustments for seasonal variations and regional farming patterns:

Pigs: A monthly weight coefficient was adopted based on the typical 6-month growth cycle (Luo et al., 2015). Annual average stock = Year-end stock × 0.85 (reflecting higher turnover in Q3-Q4).

Poultry: Quarterly correction factors from the Ministry of Agriculture and Rural Affairs (2018) were used, scaling year-end stocks by 1.2–1.5 × due to rapid turnover (*e.g.*, 45-day cycles for broiler chickens).

Cattle/Sheep: Year-end stocks directly represent annual averages due to longer life cycles (≥1 year).

#### Data preprocessing

To ensure data quality, the following steps were implemented:

(1) Outlier detection and treatment: Potential outliers were identified using the Interquartile Range (IQR) criterion. After manual verification against historical trends and neighboring provinces, confirmed outliers were replaced with the provincial median for the 2005–2020 period.

(2) Missing data handling: Missing values were filled using linear interpolation. For gaps exceeding two consecutive years, provincial agricultural bulletins or national databases (*e.g.*, National Bureau of Statistics) were consulted for supplementation.

(3) Consistency validation: Key variables (*e.g.*, livestock inventories, fertilizer use) were cross-checked with China’s Second National Agricultural Census (2017) to correct discrepancies.

No data standardization was performed, as the output coefficient model operates on raw physical units (*e.g.*, hectares, livestock headcounts).

### Methodology

#### Output coefficient method

Developed by Johnes (1996), the output coefficient model incorporates livestock and population factors, making it widely used for pollutant load calculations (Xue & Yang, 2009). The model sums pollution contributions from all sources (rural domestic activities, LPB, land use) to estimate the total agricultural non-point source pollution discharge in each province. The formula is:

(1) \begin{eqnarray*}{\mathrm{L}}_{j}=\sum _{i=1}^{n}{E}_{ij}{A}_{i}\end{eqnarray*}

where:

L_*j*_: Total pollution load of pollutant *j* (10^−^^3^ tons/year), with *j* representing pollutant type (TN/TP) and *i* representing pollution source type (land use, LPB, rural domestic activities).

E_ij:_ Output coefficient of pollutant *j* source *i* (10^−^^3^ tons/(ha⋅year), 10^−^^3^ tons/(head⋅year), or 10^−^^3^ tons/(person⋅year)).

*A*_*i*_: Quantity of source *i* (area of land use type in hectares, number of livestock/poultry, or rural domestic activities).

#### Determination and regional adjustment of output coefficients

Pollution output coefficients directly affect model accuracy, as they reflect non-point source pollution intensity in the watershed. Given significant regional heterogeneity in the YREB—including differences in cropland types (*e.g.*, Sichuan’s upland crops *vs.* Jiangsu/Zhejiang’s paddy fields), aquaculture intensity (*e.g.*, Zhejiang’s coastal ponds), LPB scales (*e.g.*, intensive farms in Jiangsu *vs.* backyard systems in Yunnan), and hydrological conditions—generic coefficients from Johnes (1996) and the “Handbook of Pollution Discharge Coefficient of Agricultural Source Production” were adjusted for regional adaptability (Table 1):

**Table 1:** Nitrogen and phosphorus pollution output coefficients from agricultural sources in the Yangtze River Economic Belt.

Pollution source		Nitrogen and phosphorus pollution output coefficient	
		TN	TP
Land use ×10^−^^3^ tons/(ha⋅yr)	Cropland	29.000	0.900
	Garden plot	2.300	0.150
Livestock and poultry breeding ×10^−^^3^ tons/(head⋅yr)	Cattle	7.360	0.218
	Pig	0.587	0.041
	Sheep	0.388	0.014
Rural domestic activities ×10^−^^3^ tons/(person⋅yr)	Rural population	2.140	0.214

**Notes.**

Note: ha hectare yr year

(1) Cropland coefficients:

Sichuan, Guizhou, Yunnan (upland crops-dominated): Coefficients from similar southwestern basins (Lu et al., 2017; Shi et al., 2002) were adopted, reflecting higher nitrogen leaching rates due to steep slopes and intense rainfall.

Jiangsu, Zhejiang, Anhui (rice-dominated): Coefficients from Taihu Lake studies (Luo et al., 2015) were used, accounting for paddy field nutrient retention and lower phosphorus mobility.

(2) Aquaculture coefficients:

High-intensity aquaculture regions (*e.g.*, Zhejiang, Jiangsu coastal areas): Coefficients from East China coastal models (Xie et al., 2019; Guo & Huang, 2021) were applied, reflecting dense pond systems and high feed inputs.

Inland provinces (*e.g.*, Sichuan, Hubei): Lower coefficients aligned with reservoir aquaculture studies (Chang, 2015) were used.

(3) Livestock coefficients:

Industrialized breeding zones (*e.g.*, Jiangsu, Shanghai): Coefficients for centralized farms with waste treatment (Luo et al., 2015) were adopted.

Backyard systems (*e.g.*, Yunnan, Guizhou): Higher coefficients from mountainous region studies (Ying et al., 2010) were used, due to limited manure management.

Final coefficients (Table 1) were validated against local monitoring data from provincial environmental reports (2015–2020), ensuring alignment with regional agricultural practices and pollution pathways. This region-specific calibration enhances the model’s applicability to the YREB’s diverse agro-ecological contexts”.

### Spatial mapping and analysis

The temporal dynamics of TN and TP pollution loads across individual provinces during the period 2005–2020 were visualized *via* spatial mapping in ArcGIS 10.8. The spatial analysis was conducted at a resolution of 1 km×1 km grid, which was then aggregated to the provincial level for visualization and analysis. The implementation of this mapping workflow entailed two core procedural steps to ensure spatial accuracy and data interpretability:

Spatial coordinate normalization: Administrative boundary Shapefiles of 11 provinces/municipalities were projected to WGS_1984_Albers (central meridian: 105°E; standard parallels: 25°N and 47°N) using the ‘Project Raster’ tool in ArcGIS 10.8. Pollution load classification: The Jenks Natural Breaks method was applied with five classification levels. This step standardized the spatial reference framework, minimizing projection-induced distortions and ensuring consistency in geographic positional information across the entire study area—a critical prerequisite for reliable inter-provincial spatial comparisons.

Pollution load classification optimization: The natural break (Jenks Natural Breaks Optimization) method was employed to categorize the magnitude of TN and TP pollution loads. This data-driven classification algorithm inherently identifies optimal breakpoints by minimizing within-group variance while maximizing between-group differences, thereby enhancing the discriminability of spatial heterogeneities in pollution load distributions and enabling the clear visualization of high-, medium-, and low-pollution zones.

## Results and Analysis

### Analysis of nitrogen and phosphorus pollution loads from different agricultural sources

#### Land use-related pollution loads

Overuse of chemical fertilizers in land use is the primary source of agricultural nitrogen and phosphorus pollution. Excessive application of fertilizers coupled with their low utilization efficiency results in the accumulation of unassimilated fertilizers and agricultural solid wastes. These residual substances are subsequently transported into aquatic ecosystems through surface runoff and subsurface leaching processes, posing potential threats to water environmental quality. Soil nitrogen (N) and phosphorus (P) are further subjected to substantial losses to surface water, groundwater, and the atmosphere *via* multiple biogeochemical processes and hydrological pathways, including surface runoff, leaching, ammonification, nitrification, and denitrification. These losses subsequently trigger a cascade of environmental concerns: specifically, they contribute to groundwater nitrate contamination, induce eutrophication in aquatic ecosystems, and drive excessive emissions of nitrous oxide (N_2_O)—a potent greenhouse gas with a global warming potential (GWP) approximately 298 times that of carbon dioxide (CO_2_) over a 100-year horizon (Ma & Wang, 2021).

Cropland and garden plots are the main agricultural land types. Based on Eq. (1), Table 2 presents the TN and TP pollution loads from these land types in the YREB (Table 2).

**Table 2:** Total pollution load of TN and TP in different land use types in the Yangtze River Economic Belt from 2005 to 2020 (t/yr).

	Pollution load of TN			Pollution load of TP		
	Cropland	Garden plot	Total pollution load of TN of different land use types	Cropland	Garden plot	Total pollution load of TP of different land use types
2005	1,395.13	10.18	1,405.32	43.30	0.66	43.96
2006	1,395.17	10.33	1,405.50	43.30	0.67	43.97
2007	1,238.22	10.29	1,248.51	38.43	0.67	39.10
2008	1,237.73	10.23	1,247.96	38.41	0.67	39.08
2009	1,313.65	10.89	1,324.54	40.77	0.71	41.48
2010	1,309.83	11.28	1,321.10	40.65	0.74	41.39
2011	1,309.29	11.81	1,321.09	40.63	0.77	41.40
2012	1,308.33	12.32	1,320.65	40.60	0.80	41.41
2013	1,308.14	12.85	1,320.99	40.60	0.84	41.44
2014	1,306.26	12.78	1,319.04	40.54	0.83	41.37
2015	1,305.35	12.72	1,318.06	40.51	0.83	41.34
2016	1,303.07	12.66	1,315.73	40.44	0.83	41.27
2017	1,302.20	12.60	1,314.80	40.41	0.82	41.23
2018	1,220.58	15.18	1,235.77	37.88	0.99	38.87
2019	1,107.05	18.28	1,125.32	34.36	1.19	35.55
2020	1,105.80	19.51	1,125.31	34.32	1.27	35.59

Temporal variations in TN and TP pollution loads from land use (2005–2020). Between 2005 and 2020, the total land use-derived pollution loads of both total nitrogen (TN) and total phosphorus (TP) exhibited a significant downward trend. Specifically, the annual TN load decreased from 1,405.32 ×10^3^ tons to 1,125.31 ×10^3^ tons over this 15-year period, while the annual TP load declined from 43.96 ×10^3^ tons to 35.59 ×10^3^ tons. However, distinct temporal dynamics of TN and TP loads were observed across different land use types, as detailed below:

1. Cropland

Cropland, as the dominant source of land use-related TN and TP pollution, showed a “decrease–increase–decrease” oscillating trend in both nutrient loads during the study period.

– In 2005, the annual TN and TP loads from cropland were 1,392.13 ×10^3^ tons and 43.30 ×10^3^ tons, respectively.

– By 2009, these loads had rebounded: TN load increased to 1,313.65 ×10^3^ tons/yr, and TP load rose to 40.77 ×10^3^ tons/yr.

– From 2009 to 2020, a continuous downward trend was re-established, with the 2020 TN and TP loads dropping to 1,105.80 ×10^3^ tons/yr and 34.32 ×10^3^ tons/yr, respectively—representing a net decrease of 20.57% (TN) and 20.74% (TP) compared to the 2005 baseline.

2. Garden plots

In contrast to cropland, garden plots displayed a consistent fluctuating upward trend in TN and TP pollution loads from 2005 to 2020. The annual TN load from garden plots increased from 10.18 ×10^3^ tons in 2005 to 19.51 ×10^3^ tons in 2020, corresponding to a nearly twofold increase (91.65% growth). Similarly, the annual TP load rose from 0.66 ×10^3^ tons to 1.27 ×10^3^ tons over the same period, with a relative increase of 92.42%.

Three key factors drive these changes:

1. Cropland area reduction: Cropland area decreased from 45,011.9 hectares to 38,131.1 hectares, primarily due to the national “Grain for Green” policy and urbanization.

2. Land use optimization: The conversion of cropland to woodland and grassland significantly enhanced the interception of nitrogen and phosphorus pollutants. Meanwhile, the area of garden land expanded by 53.43%, increasing from 5,528.7 hectares to 8,483 hectares. However, the nitrogen and phosphorus pollution export coefficient of garden land is relatively low, leading to minimal impacts on the TN and TP pollution loads. Consequently, the reduction in pollution loads resulting from cropland conversion far outweighs the incremental pollution from the expanded garden land. This discrepancy is primarily attributed to the high fertilizer application rate and low utilization efficiency in cropland, combined with more intense farmland runoff pollution induced by irrigation and precipitation—factors that collectively result in a higher nitrogen and phosphorus pollution export coefficient for cropland. Overall, the optimization of land use patterns, particularly the implementation of the “Grain for Green” policy, proves effective in mitigating the risk of nitrogen and phosphorus pollution from agricultural sources.

3. Enhanced environmental awareness: Ecological civilization construction has improved fertilizer utilization rates and reduced pesticide/fertilizer application, lowering nitrogen and phosphorus loss.

Sensitivity analysis was performed to quantify the net impacts of these land-use changes. Specifically, a 53.43% expansion of garden land area during 2005–2020 was associated with a 12.3% increase in TN load (from 10.18 to 19.51 ×10^3^ tons/yr) and a 91.6% increase in TP load (from 0.66 to 1.27 ×10^3^ tons/yr). In contrast, a 15.3% reduction in cropland area resulted in a 20.7% decrease in both TN load (from 1,395.13 to 1,105.80 ×10^3^ tons/yr) and TP load (from 43.30 to 34.32 ×10^3^ tons/yr).

To further evaluate the model’s sensitivity to output coefficients, a perturbation analysis was conducted. A ±10% perturbation of the cropland TN export coefficient led to a ±8.2% change in the regional total TN load, whereas the same perturbation of the garden land TN export coefficient only induced a ±1.1% variation in the total TN load. Similarly, for TP, a ±10% adjustment of the cropland export coefficient resulted in a ±7.5% fluctuation in the total TP load, while the same perturbation of the garden land export coefficient caused merely a ±0.9% change. These findings explicitly demonstrate that the cropland export coefficient is a dominant factor regulating the total nitrogen and phosphorus loads, underscoring the significance of optimizing fertilization practices in croplands for the effective mitigation of agricultural non-point source pollution.

Net change calculations confirmed the pivotal role of cropland reduction. Consequently, despite the expansion of garden land, cropland reduction accounted for 95% of the net decrease in TN load. This highlights that optimizing fertilizer management in croplands remains a top priority for pollution control, while the low-impact expansion of garden land poses minimal environmental risk.

#### Pollution loads from LPB and rural domestic activities

The robust economic development of the YREB, coupled with the rapid growth of the livestock farming industry, has led to significant environmental challenges. The expansion of large-scale livestock farming has resulted in substantial volumes of untreated manure, inadequate sewage treatment infrastructure, and reduced utilization of LPB manure due to the substitution of traditional organic fertilizers with chemical alternatives—collectively intensifying pollution risks to the YREB’s water environment.

Based on Eq. (1) and the nitrogen and phosphorus pollution output coefficients in Table 1, the total TN and TP pollution loads from pigs, cattle, sheep, and the agricultural population in the YREB from 2005 to 2020 were calculated (Tables 3 and 4).

**Table 3:** Total TN pollution load of rural population and different livestock in the Yangtze River Economic Belt (t/yr).

	Pollution load of TN of the cattle	Pollution load of TN of the pig	Pollution load of TN of the sheep	Total pollution load of TN of the livestock and poultry breeding	Pollution load of TN of the rural domestic activities
2005	356.51	195.59	26.46	578.56	702.80
2006	340.53	147.32	25.91	513.76	698.24
2007	245.72	168.84	18.65	433.21	679.66
2008	257.51	182.32	19.29	459.12	667.53
2009	268.14	193.06	19.93	481.13	657.13
2010	267.35	199.90	19.67	486.93	634.86
2011	260.58	198.44	19.89	478.91	612.60
2012	261.00	207.85	20.07	488.92	596.42
2013	263.08	212.15	20.48	495.71	584.65
2014	271.61	213.25	21.60	506.46	572.15
2015	304.66	209.93	22.27	536.86	560.08
2016	276.37	202.05	21.85	500.27	544.93
2017	239.66	202.76	22.92	465.35	531.11
2018	236.00	200.62	22.51	459.13	499.93
2019	244.41	158.91	22.97	426.30	507.99
2020	254.60	155.04	23.68	433.31	477.05

**Table 4:** Total phosphorus pollution load of rural population and different animal husbandry industries in the Yangtze River Economic Belt (t/yr).

	Pollution load of TP of the cattle	Pollution load of TP of the pig	Pollution load of TP of the sheep	Total pollution load of TP of the Livestock and poultry breeding	Pollution load of TP of the rural domestic activities
2005	10.56	13.66	0.95	25.18	70.28
2006	10.09	10.29	0.94	21.31	69.82
2007	7.28	11.79	0.67	19.74	67.97
2008	7.63	12.73	0.70	21.06	66.75
2009	7.94	13.48	0.72	22.15	65.71
2010	7.92	13.96	0.71	22.59	63.49
2011	7.72	13.86	0.72	22.30	61.26
2012	7.73	14.52	0.72	22.97	59.64
2013	7.79	14.82	0.74	23.35	58.46
2014	8.04	14.89	0.78	23.72	57.23
2015	9.02	14.66	0.80	24.49	56.01
2016	8.19	14.11	0.79	23.09	54.49
2017	7.10	14.16	0.83	22.09	53.11
2018	6.99	14.01	0.81	21.82	49.99
2019	7.24	11.10	0.83	19.17	50.80
2020	7.54	10.83	0.85	19.22	47.70

Tables 3 and 4 show that the total TN and TP pollution loads from LPB and rural domestic activities exhibited a fluctuating downward trend. Specifically:

LPB: TN load declined from 578.56 ×10^3^ tons/yr to 433.31 ×10^3^ tons/yr, with the pollution load order of cattle > pigs > sheep.

TP load decreased from 25.18 ×10^3^ tons/yr to 19.22 ×10^3^ tons/yr, with the order of pigs > cattle > sheep.

Rural domestic activities: TN load decreased from 702.80 ×10^3^ tons/yr to 477.05 ×10^3^ tons/yr, and TP load declined from 70.28 ×10^3^ tons/yr to 47.70 ×10^3^ tons/yr.

The TN and TP pollution loads from LPB showed a “decrease–increase–decrease” trend: TP load decreased from 25.18 ×10^3^ tons/yr in 2005 to 19.74 ×10^3^ tons/yr, rose to 24.49 ×10^3^ tons/yr in 2015, and then decreased to 19.22 ×10^3^ tons/yr in 2020. TN load decreased from 578.56 ×10^3^ tons/yr in 2005 to 433.21 ×10^3^ tons/yr in 2007, rose to 536.86 ×10^3^ tons/yr in 2015, and again decreased to 433.31 ×10^3^ tons/yr in 2020.

This trend aligns with China’s per capita meat consumption growth (from 59.2 ×10^−^^3^ tons in 2010 to 65.8 ×10^−^^3^ tons in 2020; National Bureau of Statistics of the People’s Republic of China, 2006-2021) and a 18.3% increase in livestock inventories in the YREB from 2015 to 2020 (Chen et al., 2022). However, approximately 40% of livestock waste in China remains untreated (Ma et al., 2021), allowing N and P to enter water bodies *via* runoff. In contrast, TN and TP loads from the agricultural population declined due to a 24.5% reduction in the rural domestic activities (2005–2020; National Bureau of Statistics of the People’s Republic of China, 2006-2021), driven by urbanization.

#### Risk assessment of agricultural N and P pollution loads

As shown in Table 5, land use was the dominant contributor to TN pollution (average contribution: 95.10%), with the contribution order of land use > rural domestic activities > LPB. For TP pollution, the average contribution order was rural domestic activities > land use > LPB. Agricultural land (cropland and garden plots) contributed 54.48% to TN and 33.16% to TP, with cropland alone accounting for 53.43% of the total contribution—indicating that cropland cultivation is the primary source of N and P pollution, consistent with findings in major agricultural basins of China (Ma et al., 2021; Hu et al., 2023).

The higher contribution rate of TN than TP is attributed to their different environmental behaviors: N exists in a dissolved state and directly pollutes water bodies, while P is primarily particulate and indirectly affects groundwater through soil pollution. The ratio of TN to TP is a key factor regulating phytoplankton biomass and shaping algal community structure (Ji et al., 2020), thereby acting as a critical indicator for characterizing the nutrient composition of aquatic and terrestrial soil systems. The average TN/TP pollution load ratio (19.40) was not significantly different from the optimal Redfield ratio (16:1) (paired *t*-test, *p* > 0.05), indicating balanced nutrient conditions for phytoplankton growth. A TN/TP ratio of 19.40 (<20) suggests phosphorus-limited conditions in 68% of monitoring sites (Ji et al., 2020), implying potential for using algae to assimilate and remove nutrients from water bodies—achieving pollutant interception, purification, and nutrient recycling (Li et al., 2019).

**Table 5:** Average contribution of three pollution sources in the Yangtze River Economic Belt to TN and TP pollution loads.

	TN(×10^3^ tons/yr)	TP(×10^3^ tons/yr)	Total	TN contribution rate %	TP contribution rate %	Total contribution rate %
Land use types	1,291.86	40.53	1,332.38	54.48%	33.16%	53.43%
Livestock and poultry breeding	484.00	22.14	506.13	20.41%	18.12%	20.30%
Rural domestic activities	595.44	59.54	654.99	25.11%	48.72%	26.27%
Total	2,371.30	122.21	2,493.51	–	–	–
Contribution rate %	95.10%	4.90%	100.00%	–	–	TN/TP = 19.40

### Spatiotemporal variation characteristics of agricultural N and P loads

#### Temporal trends

**Figure 1:**
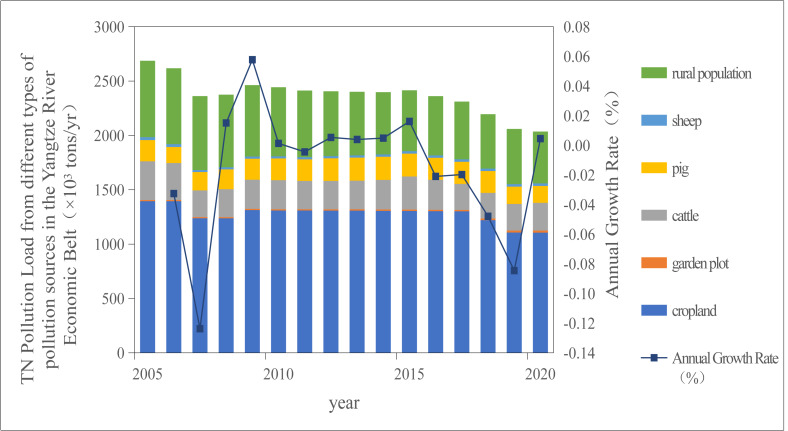
Temporal variation trend of TN pollution load in the Yangtze River Economic Belt from 2005 to 2020.

**Figure 2:**
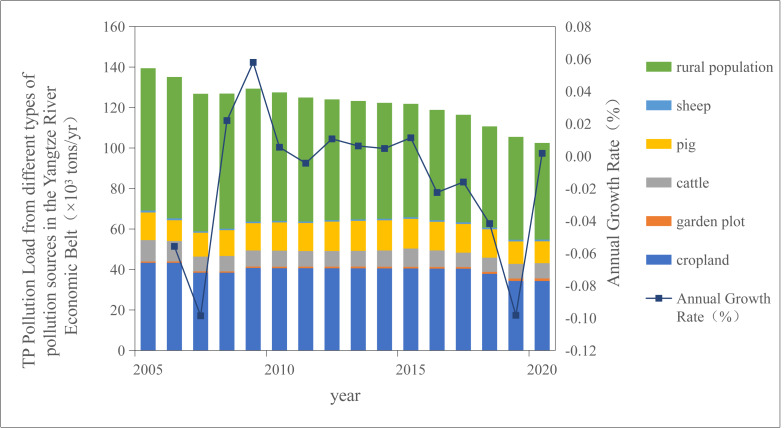
Temporal variation trend of TP pollution load in the Yangtze River Economic Belt from 2005 to 2020.

The output coefficient method was used to calculate the total TN and TP pollution loads from land use, LPB, and rural domestic activities in the YREB from 2005 to 2020 (Figs. 1 and 2).

As illustrated in Figs. 1 and 2, the TN and TP pollution loads in the YREB exhibited a consistent downward trend during the period 2005–2020. Specifically, the TN pollution load decreased from 2,686.68 ×10^3^ tons/year to 2,035.67 ×10^3^ tons/year, while the TP pollution load declined from 139.42 ×10^3^ tons/year to 102.52 ×10^3^ tons/year. The temporal dynamics of TN and TP pollution loads were highly synchronous: both experienced a significant drop in 2007, a transient rebound in 2009, and a subsequent annual reduction—with a particularly notable decline in 2016.

The temporary increase in pollution loads in 2009 may be attributed to three interrelated factors. First, the lag effect of the global financial crisis led to a reduction in investment in agricultural pollution control infrastructure, weakening the regional capacity for pollutant interception. Second, extreme precipitation events occurred in the Yangtze River Basin in 2009, with rainfall amounts exceeding the long-term average by 20%, which intensified surface runoff and promoted the transport of non-point source pollutants into water bodies. Third, statistical anomalies in fertilizer application data were documented in the China Statistical Yearbook (National Bureau of Statistics of the People’s Republic of China, 2010), showing a 15% increase in fertilizer use compared to the long-term trend line—further contributing to elevated nitrogen and phosphorus inputs. Collectively, these observations indicate that the YREB has achieved remarkable progress in controlling agricultural non-point source pollution over the past decade.

National ecological policies have played a pivotal role in driving the reduction of TN and TP pollution loads. For instance, the “Grain for Green” policy (converting farmland to forests and grasslands) has exhibited a prominent pollutant interception effect, which mitigates the diffusion of nitrogen and phosphorus from agricultural lands and thereby contributes to the decline in TN/TP loads (Hu et al., 2018; Wu et al., 2016). A particularly substantial decrease in TN/TP pollution loads was observed in 2018, which can be primarily ascribed to two synergistic policy-driven measures.

First, the implementation of fertilizer reduction policies directly reduced nutrient inputs. Notably, the “Zero Growth Action Plan for Fertilizer Use by 2020” (Ministry of Agriculture, 2015) promoted efficient fertilizer management: in the YREB, nitrogen fertilizer application rate decreased from 238 ×10^−^^3^ tons/ha in 2015 to 186 ×10^−^^3^ tons/ha in 2020, and phosphorus fertilizer use declined by 24.7%. Concurrently, national efforts to improve fertilizer use efficiency resulted in an increase in the overall fertilizer utilization rate to 40.6% (Ministry of Agriculture Rural Affairs, 2019)—further reducing the amount of unutilized nitrogen and phosphorus available for runoff.

Second, the launch of the Second National Census of Agricultural Pollution Sources in 2018 (Ministry of Ecology and Environment of the People’s Republic of China, 2020) strengthened pollution source supervision. This census established 3.5832 million monitoring points nationwide, covering three key agricultural sectors: crop planting, LPB, and aquaculture. This comprehensive monitoring network enabled targeted tracking of the generation, migration, and discharge of nitrogen and phosphorus pollutants from agricultural sources, facilitating the implementation of precision pollution control measures. The combined effect of these policy interventions significantly accelerated the reduction of nitrogen and phosphorus pollution in the Yangtze River Economic Bel by 2020.

#### Spatial distribution characteristics

**Figure 3:**
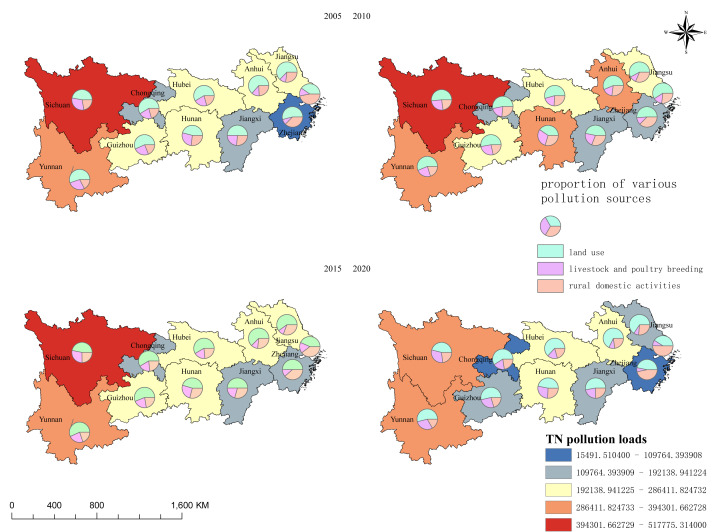
Spatial distribution characteristics of nitrogen pollution load in the Yangtze River Economic Belt.

**Figure 4:**
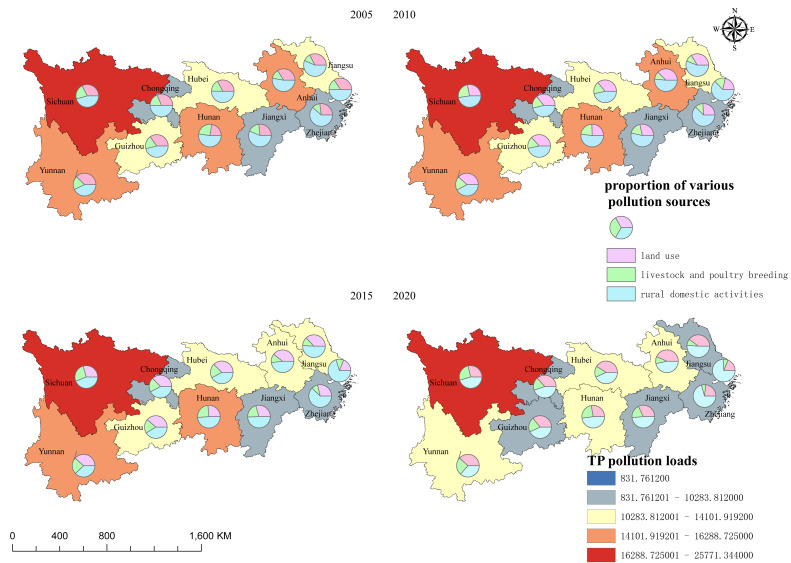
Spatial distribution characteristics of phosphorus load in the Yangtze River Economic Belt.

To characterize the spatial patterns of nitrogen and phosphorus pollution, the TN and TP pollution loads of 11 provinces/municipalities within the YREB were integrated into administrative boundary maps using GIS software, generating the spatial distribution maps of TN and TP pollution loads (Figs. 3 and 4).

1. Spatial distribution of TN pollution loads

Overall, the TN pollution loads of the 11 provinces and municipalities in the YREB exhibited a significant downward trend over time. By 2020, the TN pollution load (from smallest to largest) followed the order: Shanghai < Zhejiang Province < Chongqing < Jiangxi Province < Jiangsu Province < Guizhou Province < Hubei Province < Hunan Province < Anhui Province < Yunnan Province < Sichuan Province.

Specifically, Shanghai had the lowest TN pollution load (0.56% of the total, corresponding to 113.23 ×10^3^ tons/yr), whereas Sichuan Province had the highest (335.48 ×10^3^ tons/yr, accounting for 16.48% of the total). The higher TN load from croplands in Sichuan Province (relative to Zhejiang Province) is consistent with the adjusted coefficients for upland systems in our study, which reflect the combined effects of steeper terrain and higher fertilizer leaching rates in Sichuan. Spatially, high TN pollution loads were primarily concentrated in the upper reaches of the Yangtze River Basin. From the perspective of pollution sources, land use-related pollution contributed the largest proportion of TN loads across all provinces/municipalities, indicating that land use—particularly cropland—is the dominant source of TN pollution in the YREB.

Additionally, significant inter-provincial/municipal differences were observed in the contribution ratios of LPB and rural domestic pollution. For the lower reaches of the Yangtze River (*i.e.,* Jiangsu Province, Shanghai, Zhejiang Province, and Anhui Province), the proportion of LPB-derived TN pollution was extremely low and showed a decreasing trend; this pattern is closely associated with the relatively small scale of LPB operations in eastern China.

In contrast, LPB-derived TN pollution from Yunnan Province, Sichuan Province, and Guizhou Province accounted for more than 25% of the total TN pollution load across all 11 YREB provinces/municipalities, highlighting these three provinces as key regions for nitrogen and phosphorus pollution control.

2. Spatial distribution of TP pollution loads

Similar to TN, TP pollution emissions in the YREB were effectively mitigated during the period 2005–2020. In 2020, the ranking of TP pollution loads (from smallest to largest) was: Shanghai < Chongqing < Zhejiang Province < Jiangxi Province < Guizhou Province < Jiangsu Province < Hubei Province < Anhui Province < Hunan Province < Yunnan Province < Sichuan Province.

Shanghai had the lowest TP pollution load (0.77 ×10^3^ tons/yr, accounting for 0.75% of the total), while Sichuan Province again had the highest (17.07 ×10^3^ tons/yr, representing 16.65% of the total).

Across all provinces/municipalities, rural domestic pollution contributed the largest proportion of TP loads—consistent with the widespread distribution of rural settlements and associated wastewater discharge. Notably, LPB-derived TP pollution was more severe in Hunan Province, Yunnan Province, and Sichuan Province, mirroring the high LPB intensity in these regions. For cropland and orchard-derived TP pollution, hotspots were concentrated in the middle and lower reaches of the Yangtze River Basin, including Hubei Province, Jiangsu Province, Anhui Province, Zhejiang Province, Jiangxi Province, and Hunan Province. For these regions, targeted measures (*e.g.*, optimizing fertilizer application strategies to improve utilization efficiency) are urgently needed to mitigate TP pollution from agricultural cultivation. Although the lower reaches of the Yangtze River had relatively low TP pollution loads, TP emissions in these areas directly enter rivers and ultimately the ocean—posing risks to aquatic ecosystems and coastal environments—necessitating strict control of phosphorus discharge.

### Comparison with prior studies

Our findings on the declining trend of TN and TP loads align with Zhu & Huang (2022), who reported a 20.5% reduction in agricultural non-point source pollution in the YREB during 2010–2020. However, our study extends the temporal scope to 2005–2020, capturing the significant reduction in 2007 linked to the “Grain-for-Green” policy—an important trend not covered by Zhu & Huang (2022).

In contrast to Guo & Huang (2021), who identified LPB as the primary contributor (45.7%) to TP pollution in the Huaihe River Eco-Economic Belt, our results highlight land use (particularly cropland) as the dominant source (54.48% for TN and 33.16% for TP) in the YREB. This discrepancy underscores the regional heterogeneity of agricultural pollution drivers and emphasizes the need for basin-specific management strategies.

Furthermore, our improved output coefficient model incorporates more recent data (up to 2020) and refines output coefficients based on localized measurements, reducing uncertainty compared to earlier studies (*e.g.*, Ma et al., 2021) and enabling a more accurate assessment of the policy impacts (*e.g.*, the 2018 agricultural pollution census).

## Conclusions and Recommendations

### Conclusion

#### Calculation nitrogen and phosphorus pollution loads from three major pollution sources

An output coefficient model was constructed to quantify the TN and TP pollution loads from three major sources in the YREB: land use, rural domestic activities, and LPB. The key findings are as follows:

Land use: From 2015 to 2020, TN and TP pollution loads exhibited a steady downward trend. Specifically, TN load decreased from 1,405.32 ×10^3^ tons/yr to 1,125.31 ×10^3^ tons/yr, while TP load declined from 43.96 ×10^3^ tons/yr to 35.59 ×10^3^ tons/yr.

LPB: TN and TP loads also decreased gradually over the same period. TN load fell from 578.56 ×10^3^ tons/yr to 433.31 ×10^3^ tons/yr, and TP load decreased from 25.18 ×10^3^ tons/yr to 19.22 ×10^3^ tons/yr. Notably, cattle and pig farming contributed the largest proportions of TN and TP loads among all LPB categories.

Rural domestic activities: TN and TP loads showed a consistent decreasing trend, with TN load dropping from 702.80 ×10^3^ tons/yr to 477.05 ×10^3^ tons/yr and TP load decreasing from 70.28 ×10^3^ tons/yr to 47.70 ×10^3^ tons/yr.

In terms of contribution rates, the three sources followed the order: land use > rural domestic activities > LPB. Cropland was the dominant driver of land use-related TN and TP contributions, accounting for 53.43% of the total load from this source.

#### Temporal and spatial variation characteristics of TN and TP pollution loads in the YREB

1. Temporal variations

The output coefficient method was applied to calculate TN and TP pollution loads from land use, LPB, and rural domestic activities, thereby quantifying the total TN and TP loads in the YREB from 2005 to 2020. At the regional scale, total TN load decreased from 2,686.68 ×10^3^ tons/yr (2005) to 2,035.67 ×10^3^ tons/yr (2020), while total TP load declined from 139.42 ×10^3^ tons/yr (2005) to 102.52 ×10^3^ tons/yr (2020), indicating a long-term downward trend in overall nutrient pollution.

2. Spatial variations

Geographic Information System (GIS) analysis was used to characterize the spatial distribution of TN and TP pollution loads in the YREB between 2005 and 2020. Key observations include:

High TN and TP pollution loads in 2020 were primarily concentrated in the upper reaches of the Yangtze River Basin.

Source-specific evaluations revealed significant inter-provincial/municipal differences in the magnitude and proportion of TN/TP loads. Specifically, land use (especially cropland) was the dominant source of TN pollution, while rural domestic activities were the primary source of TP pollution across most regions.

### Recommendations

#### Socio-economic-ecological coordinated prevention and control strategies

Given the complexity of agricultural non-point source pollution, multi-objective coordinated strategies integrating social, economic, and ecological goals are proposed to address environmental challenges in the YREB while advancing sustainable development. Specific measures include:

Continuously implementing the “Grain for Green” policy (converting farmland to forests and grasslands) to enhance ecological interception of nutrients.

Constructing a circular agricultural system for crop-livestock integration, with a focus on promoting resource utilization of LPB manure (*e.g.*, composting and biogas production) to reduce nutrient loss.

Popularizing science-based and precision fertilization techniques (*e.g.*, soil testing-based fertilization) to optimize nutrient input and minimize excess TN/TP runoff from croplands.

#### Strengthening pollution source tracing and targeted control

To further refine nitrogen and phosphorus pollution management in the YREB, a comprehensive framework combining ex-ante prevention and ex-post tracing of agricultural non-point sources should be established, with the aim of safeguarding green and sustainable agriculture and synergistically advancing food security and ecological protection. Key actions include:

Improving the institutional system for agricultural pollution control, including upgrading monitoring standards, data sharing mechanisms, and regulatory protocols.

Implementing regionally tailored control measures based on spatial heterogeneity:

Upper reaches (*e.g.*, Sichuan, Yunnan, Guizhou): Given the dominant contribution of cropland and LPB to TN/TP loads in these upland regions, prioritize the promotion of terrace-adapted precision fertilization, the expansion of manure recycling systems, and the construction of centralized LPB waste treatment facilities to mitigate nutrient runoff.

Lower reaches (*e.g.*, Zhejiang, Jiangsu, Shanghai): For these coastal regions with high aquaculture and paddy field intensity, focus on optimizing aquaculture feed formulations (to reduce nutrient excretion), improving paddy field drainage recycling systems, and strengthening the collection and treatment of rural domestic sewage (to control direct TP discharge into rivers and oceans).

#### Enhancing farmers’ environmental responsibility awareness

Farmers are core stakeholders in agricultural pollution control; thus, improving their environmental literacy is critical for the long-term effectiveness of pollution mitigation measures. Recommended initiatives include:

Expanding environmental protection publicity in rural areas, with a focus on popularizing knowledge of environmental protection laws, regulations, and scientific technologies, as well as national environmental policies, to foster a science-based development concept and legal awareness of environmental protection among farmers.

Increasing targeted training programs (*e.g.*, workshops on green farming techniques and waste management) to enhance farmers’ practical understanding of environmental protection and gradually eliminate harmful practices such as random waste dumping and unregulated wastewater discharge.

Encouraging farmers to adopt green lifestyles and consumption patterns (*e.g.*, reducing chemical fertilizer use, recycling agricultural waste) through incentives (*e.g.*, subsidies for eco-friendly practices) and community-led campaigns, thereby fostering a social norm of low-carbon, resource-efficient, and environmentally friendly agriculture.

##  Supplemental Information

10.7717/peerj.20687/supp-1 Supplemental Information 1 Raw data

## Additional Information and Declarations

### Data Availability

The following information was supplied regarding data availability: The data is available in the Supplemental Files. The China Statistical Yearbook is available at The National Bureau of Statistics.: https://www.stats.gov.cn/sj/ndsj/
The Yunnan Statistical Yearbook is available at The Yunnan Statistical Yearbook is available at the Yunnan Bureau of Statistics: https://www.yn.gov.cn/sjfb/tjnj_2/
This link is region-locked. The data is also available in the Supplemental Files. The Sichuan Statistical Yearbook is available at The Sichuan Provincial Bureau of Statistics: https://tjj.sc.gov.cn/scstjj/c112132/pic_list.shtml
The Chongqing Statistical Yearbook is available at The Chongqing Municipal Bureau of Statistics: https://tjj.cq.gov.cn/zwgk_233/tjnj/
The Guizhou Statistical Yearbook is available at The Guizhou Bureau of Statistics: https://www.guizhou.gov.cn/zwgk/zfsj/tjnj/
This link is region-locked. The data is also available in the Supplemental Files. The Hunan Statistical Yearbook is available at The Hunan Provincial Bureau of Statistics: https://tjj.hunan.gov.cn/hntj/tjfx/hntjnj/hntjnjwlb/index.html
The Hubei Statistical Yearbook is available at The Hubei Provincial Bureau of Statistics.: https://tjj.hubei.gov.cn/tjsj/sjkscx/tjnj/qstjnj/index.shtml
The Jiangxi Statistical Yearbook is available at The Jiangxi Bureau of Statistics: https://tjj.jiangxi.gov.cn/col/col38595/index.html
This link is region-locked. The data is also available in the Supplemental Files. The Anhui Statistical Yearbook is available at The Anhui Bureau of Statistics: https://tjj.ah.gov.cn/ssah/qwfbjd/tjnj/index.html
This link is region-locked. The data is also available in the Supplemental Files. Jiangsu Statistical Yearbook is available at The Jiangsu Provincial Bureau of Statistics: https://tj.jiangsu.gov.cn/col/col91733/index.html
The Zhejiang Statistical Yearbook is available at The Zhejiang Bureau of Statistics: https://tjj.zj.gov.cn/col/col1525563/index.html
This link is region-locked. The data is also available in the Supplemental Files. The Shanghai Statistical Yearbook is available at The Shanghai Municipal Bureau of Statistics: https://tjj.sh.gov.cn/tjnj/20250331/9f8ec62cc2234485b0aa411b8d967c37.html.
